# Metabolomic Identification of a Novel, Externally Validated Predictive Test for Gestational Diabetes Mellitus

**DOI:** 10.1210/clinem/dgac240

**Published:** 2022-04-18

**Authors:** Ulla Sovio, Gemma L Clayton, Emma Cook, Francesca Gaccioli, D Stephen Charnock-Jones, Deborah A Lawlor, Gordon C S Smith

**Affiliations:** Department of Obstetrics and Gynaecology, University of Cambridge; NIHR Cambridge Biomedical Research Centre, Cambridge, UK; Centre for Trophoblast Research, Department of Physiology, Development and Neuroscience, University of Cambridge, Cambridge, UK; NIHR Bristol Biomedical Research Centre, Bristol, UK; MRC Integrative Epidemiology Unit at the University of Bristol, Bristol, UK; Population Health Sciences, Bristol Medical School, Bristol, UK; Department of Obstetrics and Gynaecology, University of Cambridge; NIHR Cambridge Biomedical Research Centre, Cambridge, UK; Department of Obstetrics and Gynaecology, University of Cambridge; NIHR Cambridge Biomedical Research Centre, Cambridge, UK; Centre for Trophoblast Research, Department of Physiology, Development and Neuroscience, University of Cambridge, Cambridge, UK; Department of Obstetrics and Gynaecology, University of Cambridge; NIHR Cambridge Biomedical Research Centre, Cambridge, UK; Centre for Trophoblast Research, Department of Physiology, Development and Neuroscience, University of Cambridge, Cambridge, UK; NIHR Bristol Biomedical Research Centre, Bristol, UK; MRC Integrative Epidemiology Unit at the University of Bristol, Bristol, UK; Population Health Sciences, Bristol Medical School, Bristol, UK; Department of Obstetrics and Gynaecology, University of Cambridge; NIHR Cambridge Biomedical Research Centre, Cambridge, UK; Centre for Trophoblast Research, Department of Physiology, Development and Neuroscience, University of Cambridge, Cambridge, UK

**Keywords:** pregnancy, metabolomics, gestational diabetes mellitus, prediction

## Abstract

**Context:**

Undiagnosed gestational diabetes mellitus (GDM) is a major preventable cause of stillbirth. In the United Kingdom, women are selected for diagnostic testing for GDM based on risk factors, including body mass index (BMI) > 30 kg/m^2^.

**Objective:**

To improve the prediction of GDM using metabolomics.

**Methods:**

We performed metabolomics on maternal serum from the Pregnancy Outcome Prediction (POP) study at 12 and 20 weeks of gestational age (wkGA; 185 GDM cases and 314 noncases). Predictive metabolites were internally validated using the 28 wkGA POP study serum sample and externally validated using 24- to 28-wkGA fasting plasma from the Born in Bradford (BiB) cohort (349 GDM cases and 2347 noncases). The predictive ability of a model including the metabolites was compared with BMI > 30 kg/m^2^ in the POP study.

**Results:**

Forty-seven predictive metabolites were identified using the 12- and 20-wkGA samples. At 28 wkGA, 4 of these [mannose, 4-hydroxyglutamate, 1,5-anhydroglucitol, and lactosyl-N-palmitoyl-sphingosine (d18:1/16:0)] independently increased the bootstrapped area under the receiver operating characteristic curve (AUC) by >0.01. All 4 were externally validated in the BiB samples (*P* = 2.6 × 10^−12^, 2.2 × 10^−13^, 6.9 × 10^−28^, and 2.6 × 10^−17^, respectively). In the POP study, BMI > 30 kg/m^2^ had a sensitivity of 28.7% (95% CI 22.3-36.0%) and a specificity of 85.4% whereas at the same level of specificity, a predictive model using age, BMI, and the 4 metabolites had a sensitivity of 60.2% (95% CI 52.6-67.4%) and an AUC of 0.82 (95% CI 0.78-0.86).

**Conclusions:**

We identified 4 strongly and independently predictive metabolites for GDM that could have clinical utility in screening for GDM.

Gestational diabetes mellitus (GDM) is pregnancy-related hyperglycemia, which usually normalizes following birth. Multiple observational studies have shown that women with GDM are more likely to have had a previous pregnancy complicated by stillbirth (1-3), indicating that undiagnosed and untreated GDM may have been a factor in the preceding loss. Moreover, a national audit of term stillbirths in the United Kingdom in 2013 identified failure to screen appropriately for GDM was 1 of the major potentially preventable elements of care (4), echoing a similar report 15 to 20 years earlier (5). There is direct evidence that screening for GDM and treating the condition reduces the risk of severe adverse outcomes of pregnancy (6, 7).

The approach to screening differs between countries. In some (eg, the United States), women generally have a biochemical screening test with a 50-g nonfasting glucose challenge test (GCT) at 24 to 28 weeks of gestational age (wkGA). Women with an elevated 1-hour post-challenge plasma glucose level are then selected for a diagnostic fasting oral glucose tolerance test (OGTT). In current UK practice, women are selected for an OGTT at ~28 wkGA based on maternal risk factors assessed in their first antenatal clinic assessment (8), including maternal prepregnancy or early pregnancy obesity [body mass index (BMI) > 30 kg/m^2^]. However, systematic reviews demonstrate poor predictive ability of such screening strategies (9).

We recently reported that metabolomic analysis of maternal serum could identify a novel blood test for fetal growth restriction that was equally predictive for large for gestational age infants (10, 11). In the present study, we applied a similar analytic approach to GDM and our aims were (1) to identify and externally validate a novel predictive test for GDM using metabolomics and (2) to compare its predictive performance with the established maternal risk factor of BMI > 30 kg/m^2^.

## Methods

### Pregnancy Outcome Prediction Study

The Pregnancy Outcome Prediction (POP) study was a prospective cohort study of unselected nulliparous women with a singleton pregnancy attending the Rosie Hospital (Cambridge, UK) between January 2008 and July 2012, and it has been previously described in detail (12, 13). Ethical approval was obtained from the Cambridgeshire 2 Research Ethics Committee (reference no. 07/H0308/163). In brief, a total of 4512 study participants were recruited at the time of their dating ultrasound at around 12 wkGA and gave written informed consent. Blood samples were collected at recruitment and at further research visits at 20, 28, and 36 wkGA. A total of 4212 women were followed from recruitment through to delivery, after which outcome data were obtained. Women were eligible for the present study if they had blood samples obtained at recruitment, 20 wkGA, and/or 28 wkGA and had maternal age and early pregnancy BMI recorded. All samples were stored at −80°C from phlebotomy to analysis. The mean storage time of the samples from the women eligible for the present study was 5.4 years (range 3.4-8.2 years).

The diagnosis of GDM in the POP study was based on routine clinical care and was defined using a 75-g fasting OGTT applying diagnostic thresholds adapted from the World Health Organization (2008-2010) or the criteria adapted from the International Association of Diabetes and Pregnancy Study Groups (2011 onward), as previously described (14). Women were selected for the 75-g fasting OGTT based on the result of a 50-g nonfasting GCT at 28 weeks. The POP study samples were analyzed as a case-cohort design (15). We studied 185 cases of GDM, irrespective of the method of treatment (drug and/or dietary), and results were compared with a random sample of the cohort of 314 women who did not have a clinical diagnosis of GDM [see Supplemental Figure 1 (16) for selection].

### Born in Bradford Study

The Born in Bradford (BiB) study was conducted between 2007 and 2011 and has been described previously in detail (17-19). Ethical approval was obtained from the Bradford Research Ethics Committee, and all study participants gave written informed consent. In brief, 12 500 pregnant women of mixed parity and singleton or multiple pregnancies were recruited to the study. Fasting plasma samples were collected at 24 to 28 wkGA, processed within 2.5 hours, and stored at −80°C for research purposes. These were available for 85% of the invited participants (no other research blood samples were obtained in pregnancy). Women were eligible for the current study if they had both plasma and the result of a fasting 75-g OGTT performed at 24 to 28 wkGA available.

The diagnosis of GDM in the BiB study was based on an OGTT at 24 to 28 wkGA, which was routinely offered to all women, except those with preexisting diabetes [n = 67 (0.5%)]. GDM was defined using the modified World Health Organization criteria as previously described (20) and, as with the POP study, included women subsequently treated by medication and/or diet.

We studied 2 subgroups of participants from the BiB study [see Supplemental Figures 2 and 3 (16) for selection] (19). Subgroup 1 (BiB 1) was a randomly selected subcohort of 1000 women who were either White British or Pakistani origin (19). After the exclusion of 21 women without data on GDM, this included 89 women with GDM and 890 women without GDM. Subgroup 2 (BiB 2) included 1717 participants selected using a case cohort design (excluding the women of BiB 1 from the selection) (19), comprising 260 women with GDM and a random sample of 1457 BiB participants without GDM. Finally, analyses were also performed after pooling the cases (n = 349) and the noncases (n = 2347) from each of the subgroups.

### Analytic Approach

The overall approach of the study was (1) to identify independent metabolite predictors of GDM using the 12- and 20-wkGA POP study samples, (2) to validate the selected metabolites internally and develop a predictive ratio using the 28-wkGA POP study sample, and (3) to validate individual predictors and the ratio externally in the BiB cohort. We have outlined the approach in Supplemental Figure 4 (16).

### Metabolomic Analyses

Metabolites were measured by Metabolon (Morrisville, NC, USA) using an ultrahigh performance liquid chromatography-tandem mass spectroscopy platform, as previously described (10, 21). The method reported data on 837 metabolites of known identity (as of March 31, 2016), and these were the candidate predictors included in the statistical analysis. Metabolite levels were expressed as scaled imputed relative concentrations; specifically, the curve of primary mass spectrometry ions was expressed as the multiples of the median (MoM) value for all batches processed on the given day, and these were log-transformed for analysis (10). The sample type varied between the 2 studies: nonfasting maternal serum samples were analyzed from the POP study, and fasting plasma samples were analyzed from the BiB study. The Metabolon data sets were completed in March 2016 for the POP study, in December 2017 for the BiB 1, and in December 2018 for the BiB 2 (19).

### Statistical Methods

Initial identification of metabolites associated with GDM in the POP study was performed by fitting a longitudinal linear mixed model for each log-transformed metabolite (as an outcome) on GDM case-control status, using measurements from maternal serum at 12 and 20 wkGA, adjusting each model for maternal age and BMI at recruitment. The difference in the metabolite means in GDM cases and controls and the associated *P*-value from a composite Chi-squared test were estimated at 12 and 20 wkGA by fitting interaction terms between GDM and each gestational age, an approach we have used in several previous publications (10, 15, 21). Excess of low composite *P*-values at 12 and 20 wkGA was tested using a 1-sample Kolmogorov-Smirnov test against the null hypothesis of a uniform distribution of *P*-values between 0 and 1, and a frequency plot of the composite *P*-values was generated. The metabolites were then ranked by the composite *P*-value at 12 and 20 wkGA. To correct for multiple testing, false discovery rate–adjusted *P*-values (ie, *Q* values) were estimated using the Simes method (22, 23). Heatmaps were drawn based on the metabolites with *Q* < 0.05.

Subsequently, associations with GDM were quantified using area under the receiver operating characteristic curve (AUC) and by logistic regression. In the latter, odds ratios and 95% CIs were calculated for a 1SD increase in the log-transformed MoM. The metabolites with *Q* < 0.05 in the initial identification were selected for further analysis at 20 wkGA using a forward stepwise logistic regression (*P* < 0.05 for entry and *P* < 0.1 for removal). A baseline logistic model with maternal age and BMI was fitted, and independently predictive metabolites measured at 20 wkGA were identified using the forward stepwise logistic regression. Metabolites that were identified as independently predictive at 20 wkGA were then assessed on whether they improved the AUC for GDM (estimated using 1000-fold bootstrapping to correct for optimism) using the 28-wkGA metabolite measurements. These were added into the baseline logistic model 1 by 1 starting from the most informative metabolite, selecting those that increased the corrected AUC by >0.01.

We then aimed to determine whether the selected variables included both positively and negatively associated metabolites to derive a predictive ratio (10, 11) by calculating the product of the MoMs of the positively associated metabolites divided by the product of the MoMs of the negatively associated metabolites. The performance of the predictive ratio was then compared with the performance of a model where each of the metabolites included in the ratio were treated as independent variables.

The performance of the model including maternal age and BMI at 12 wkGA and the selected metabolites at 28 wkGA was then compared with the performance of the current screening method (BMI > 30 kg/m^2^) at a fixed specificity. Standard screening statistics [sensitivity, specificity, positive and negative likelihood ratio (LR), positive and negative predictive value (PPV and NPV, respectively), and diagnostic odds ratio (DOR)] were calculated from 2 × 2 tables, weighting the comparison group by the inverse of the sampling fraction where appropriate.

Statistical analyses of the POP study data were performed using Stata version 15.1. and R version 4.0.3.

### External Validation in the Born in Bradford Study

External validation of the main findings was performed according to a prespecified analysis plan based on the initial results from the POP study and finalized prior to any analysis of the BiB study data [Supplemental Panel 1 (16)]. Briefly, the metabolites, their products, and the ratio of products were analyzed as exposures in relation to GDM (primary outcome) and fasting and postload maternal plasma glucose concentration (secondary outcomes). The analyses reported data on BiB 1 and 2 independently and the pooled sample of both subgroups. Associations with the outcomes were assessed using the AUC and logistic and linear regressions, as appropriate. The external validation analyses were performed using Stata version 16.

## Results

### POP Study

A total of 185 cases of GDM and 314 noncases [see Supplemental Figure 1 for selection (16)] were analyzed using the linear mixed model, comparing metabolite values at 12 and 20 weeks. Eight xenobiotic metabolites that demonstrated minimal variation were excluded, leaving 829 metabolites of known identity for analysis. A frequency plot of the estimated *P*-values showed a statistically significant (Kolmogorov-Smirnov test *P* < 0.001) excess of small *P*-values [Supplemental Figure 5 (16)]. After applying the false discovery rate correction, 47 out of 829 metabolites [Supplemental Table 1, Supplemental Figures 6-8 (16)] were selected for further analysis. The corrected overall critical *P*-value was 0.00283, corresponding to the *Q*-value of 0.05.

Among the women who had metabolite data available at 20 wkGA (n = 469, including 171 GDM cases and 298 noncases), GDM was associated with both maternal age (AUC = 0.61, 95% CI 0.56-0.66) and BMI (AUC = 0.65, 95% CI 0.60-0.70), and the combination of the 2 in a model was moderately predictive (AUC = 0.69, 95% CI 0.64-0.74) (Table 1). Using the 20-wkGA data, forward stepwise regression of the 47 metabolites plus maternal age and BMI demonstrated that 8 metabolites were independently associated with GDM. The metabolites, their AUCs, and the correlations between the metabolites, maternal age, and BMI are tabulated (Supplemental Table 2, Supplemental Figures 9 and 10 (16)].

**Table 1.** AUC with a 95% CI in the prediction of GDM using maternal characteristics and metabolites

	AUC (95% CI)	
Predictors	20 wkGA (n = 469)	28 wkGA (n = 466)
Maternal BMI	0.65 (0.60-0.70)	0.64 (0.58-0.69)
Maternal age	0.61 (0.56-0.66)	0.63 (0.58-0.68)
Maternal BMI and age	0.69 (0.64-0.74)	0.69 (0.63-0.74)
4-metabolite ratio	0.72 (0.67-0.77)	0.75 (0.71-0.80)
4-metabolite model	0.74 (0.70-0.79)	0.80 (0.76-0.84)
4-metabolite ratio, maternal age, and BMI	0.75 (0.70-0.79)	0.78 (0.73-0.82)
4 metabolites, maternal age, and BMI	0.76 (0.72-0.81)	0.82 (0.78-0.86)

The number of women in the analyses at 20 and 28 wkGA are 469 (171 gestational diabetes mellitus cases and 298 controls) and 466 (171 gestational diabetes mellitus cases and 295 controls), respectively. Maternal BMI and age were recorded at 12 wkGA, and the number of women included in the analysis of maternal characteristics was determined by the availability of metabolite data at 20 and 28 wkGA.

Abbreviations: AUC, area under the receiver operating characteristic curve; BMI, body mass index; wkGA, weeks of gestational age.

We then tested the independent predictive ability of the 8 selected metabolites using the data from 28 wkGA (n = 466 women) [Supplemental Table 3 (16)], starting with a baseline model including maternal age and BMI. Metabolites were consecutively added to the model, starting with the most informative, and we continued to add them until the next most informative metabolite increased the AUC (corrected for overfitting by bootstrapping with 1000 replications) by <0.01. Four out of the 8 metabolites increased the bootstrapped AUC by >0.01. Two of the metabolites were positively associated with GDM (mannose and 4-hydroxyglutamate), and 2 were negatively associated [1,5-anhydroglucitol and lactosyl-N-palmitoyl-sphingosine (d18:1/16:0)]. The values at 12 to 28 wkGA are plotted in relation to the subsequent diagnosis of GDM [Supplemental Figure 11 (16)]. We then calculated a 4-metabolite ratio (ie, the product of the MoM values of the 2 positively associated metabolites divided by the product of the MoM values of the 2 negatively associated metabolites).

### BiB Study

**Figure 1.**
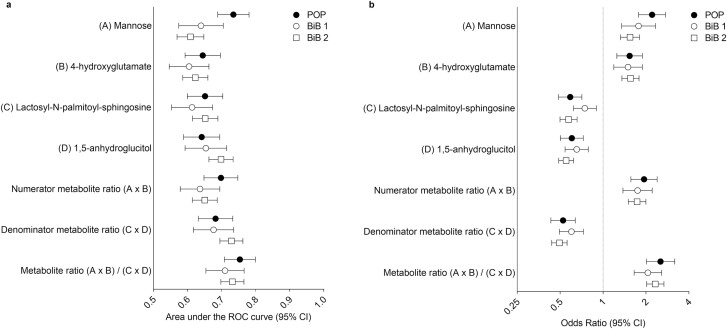
Area under the receiver operating characteristic curve (A) and odds ratio (B) for metabolite measurements at ~24-28 wkGA in relation to GDM. The POP study included 171 GDM cases and 295 controls; the BiB subgroup 1 (BiB 1) included 89 GDM cases and 890 controls, and the BiB subgroup 2 (BiB 2) included 260 GDM cases and 1457 controls. Abbreviations: BiB, Born in Bradford; GDM, gestational diabetes mellitus; POP, Pregnancy Outcome Prediction; wkGA, weeks of gestational age.

BiB 1 and 2 are described in Supplemental Tables 4 and 5 (16), respectively. All 4 metabolites, their products, and the 4 metabolite ratio were validated in these subgroups [Fig. 1; Supplemental Table 6 (16)] and when the subgroups were pooled [Supplemental Table 6 (16)]. The ratio discriminated GDM almost as well in the 24- to 28-wkGA plasma sample from the BiB study (AUC = 0.71 in BiB 1 and AUC = 0.73 in BiB 2) as in the 28-wkGA serum sample from the POP study (AUC = 0.75). One-sided *P*-values from the logistic regression for the metabolite ratio were very small, 7.8 × 10^−11^ for BiB 1 and 7.4 × 10^−33^ for BiB 2. *P*-values for the individual metabolites were also well below the validation threshold of 0.0083 [Supplemental Table 6 (16)]. Furthermore, fasting and postload glucose (secondary outcomes) as continuous variables showed strong associations with all individual metabolites, their products, and the ratio in the expected direction [Supplemental Tables 7 and 8 (16)], and for the 4-metabolite ratio, the *P*-values from the pooled analysis were 1.1 × 10^−120^ for fasting glucose and 2.4 × 10^−86^ for postload glucose. As a sensitivity analysis, we tested whether the associations with GDM were similar by ethnicity, and we did not identify any evidence of interactions (all *P*s > 0.05).

### Predictive Performance of the 4 Metabolites in the POP Study

We found that the AUC for the ratio was lower than the optimism-corrected AUC for the 4 individual metabolites when treated as independent variables in a logistic regression model. Hence, all subsequent evaluation of the 4 metabolites as a predictive test included using the 4 metabolites as independent predictive variables. The baseline model using maternal age and BMI had an AUC of 0.69 (95% CI 0.63-0.74). The ratio on its own performed better than the model using age and BMI alone (Table 1) at both 20 wkGA (AUC = 0.72) and at 28 wkGA (AUC = 0.75). A model combining the ratio at 28 wkGA with maternal characteristics gave an AUC of 0.78. The strongest prediction of GDM was achieved by a model that included the 4 metabolites separately at 28 wkGA and the maternal characteristics (AUC = 0.82).

**Figure 2.**
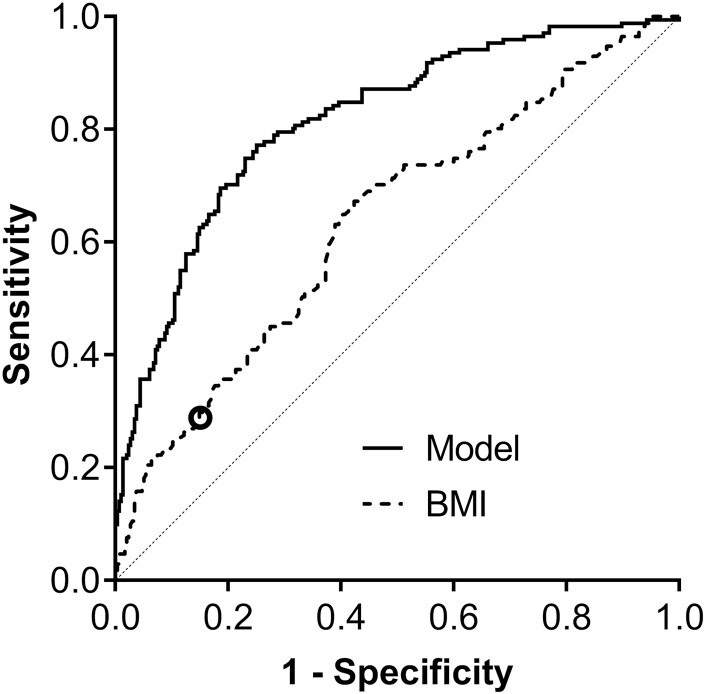
Comparison of the model and BMI alone for the discrimination of GDM cases and controls. The model included maternal BMI and age at 12 wkGA and the 4 metabolites measured at 28 wkGA. The analysis included 171 GDM cases and 295 controls. Area under the receiver operating characteristic curve (AUC; 95% CI) = 0.82 (0.78-0.86) and 0.64 (0.58-0.69), respectively, 2-sided DeLong test *P* < 0.0001 for the AUC comparison. The diagonal line represents the AUC of 0.5 (no discrimination). The 𝝤 symbol indicates BMI = 30 kg/m^2^. At this threshold, the specificity of BMI as a screening test was 85.4%, and the sensitivity was 28.7%. With the same specificity, the model resulted in a sensitivity of 60.2%. Abbreviations: AUC, area under the receiver operating characteristic curve; BMI, body mass index; GDM, gestational diabetes mellitus; wkGA, weeks of gestational age.

As BMI > 30 kg/m^2^ is currently used to screen women for the risk of GDM, we compared receiver operating characteristic plots of early pregnancy BMI and the model including the 4 metabolites measured at 28 wkGA and the maternal characteristics (Fig. 2). At the threshold of BMI = 30, the specificity of BMI as a screening test was 85.4%. We compared the screening statistics for BMI and for the model using the same threshold of specificity. The cutoff of 47% predicted probability of GDM from the model resulted in the specificity of 85.4%. The model using age, BMI, and the 4 metabolites as independent predictors performed better than BMI in screening for GDM (Table 2) at this level of specificity. The sensitivity was over 2-fold higher [60.2% (95% CI 52.6-67.4%) vs 28.7% (95% CI 22.3-36.0%)] as was the positive LR, and the DOR was 3.5-fold higher. The PPV and NPV were also higher for the model-based screening test compared with maternal BMI > 30 kg/m^2^ (PPV: 15.7% vs 8.1%, respectively; NPV: 97.9% vs 96.4%, respectively). Additionally, we calculated screening statistics using a threshold that resulted in ~5% screen positive rate in the random subsample (cutoff of predicted probability of GDM from the model = 73%). This resulted in the same sensitivity of 28.7% as BMI > 30 kg/m^2^ but in a much higher specificity (96.6%), positive LR (8.5), PPV (27.6%), and DOR (11.4) (Table 2). We repeated the comparisons using the model that included the 4 metabolites measured at 20 wkGA and BMI and age (Table 2). Although the diagnostic effectiveness of the test was reduced compared with 28 wkGA, the associations were still stronger than for BMI alone: women in the top 5% by the model had an absolute risk of a diagnosis of GDM of >20% compared with 9% for obesity.

**Table 2.** Diagnostic effectiveness of screening for gestational diabetes mellitus using the current method (BMI > 30 kg/m^2^) vs the model including 4 metabolites at 28 wkGA or 20 wkGA and BMI and age at 12 wkGA

Screening test	TP/FP	TN/FN	Screen + Comp^a^	Positive LR (95% CI)	Negative LR (95% CI)	Sensitivity (95% CI)	Specificity (95% CI)	PPV^a^ (95% CI)	NPV^a^ (95% CI)	DOR (95% CI)
28 wkGA (n = 466)										
BMI > 30 kg/m^2^	49/43	252/122	15.4	2.0 (1.4-2.8)	0.84 (0.75-0.93)	28.7 (22.3-36.0)	85.4 (80.9-89.0)	8.1 (5.5-11.9)	96.4 (95.5-97.1)	2.4 (1.5-3.7)
P(GDM) > 47%	103/43	252/68	16.7	4.1 (3.1-5.6)	0.47 (0.38-0.56)	60.2 (52.6-67.4)	85.4 (80.9-89.0)	15.7 (11.5-21.1)	97.9 (97.3-98.4)	8.9 (5.7-13.7)
P(GDM) > 73%	49/10	285/122	4.9	8.5 (4.4-16.2)	0.74 (0.67-0.81)	28.7 (22.3-36.0)	96.6 (93.8-98.2)	27.6 (15.9-43.4)	96.8 (96.0-97.4)	11.4 (5.6-23.1)
20 wkGA (n = 469)										
BMI > 30 kg/m^2^	52/40	258/119	14.3	2.3 (1.6-3.3)	0.80 (0.72-0.90)	30.4 (23.9-37.8)	86.6 (82.2-90.0)	9.2 (6.2-13.3)	96.5 (95.7-97.2)	2.8 (1.8-4.5)
P(GDM) > 51%	85/40	258/86	14.9	3.7 (2.7-5.1)	0.58 (0.50-0.68)	49.7 (42.2-57.2)	86.6 (82.2-90.0)	14.2 (10.2-19.5)	97.5 (96.8-98.0)	6.4 (4.1-9.9)
P(GDM) > 67%	41/11	287/130	4.9	6.5 (3.4-12.3)	0.79 (0.72-0.86)	24.0 (18.1-31.0)	96.3 (93.4-98.0)	22.5 (12.7-36.6)	96.6 (95.8-97.2)	8.2 (4.1-16.5)

Maternal BMI was recorded at 12 wkGA, and the number of women included in the analysis of maternal characteristics was determined by the availability of metabolite data at 20 and 28 wkGA. The total number of women in the analysis at 28 wkGA was 466, including 171 cases of GDM and 295 controls, and at 20wkGA, it was 469, including 171 cases and 298 controls.

Abbreviations: BMI, body mass index; Comp, comparator group; DOR, diagnostic odds ratio; FN, false negative; FP, false positive; LR, likelihood ratio; NPV, negative predictive value; P(GDM), predicted probability of gestational diabetes mellitus from the model, selected to match the specificity of the screening test BMI > 30 kg/m^2^ or to achieve the screen positive rate of ~5%; PPV, positive predictive value; TN, true negative; TP, true positive; wkGA, weeks of gestational age.

^a^Due to the case cohort design, the proportion of screen positives was calculated in the comparator group (ie, the random subcohort) among women who had the metabolite measurements available (at 28 wkGA, n = 306 including 11 cases of GDM and 295 noncases, and at 20 wkGA, n = 308 including 10 cases of GDM and 298 noncases), and PPV and NPV were weighted by the inverse of the random subcohort sampling fraction. The proportions of screen positives, sensitivity, specificity, PPV, and NPV are given as percentages.

Finally, we determined the association between the ratio at 28 wkGA and the birth weight z-score in women without a clinical diagnosis of GDM in the POP study. We observed that higher levels of the ratio were associated with higher mean birth weight [Supplemental Figure 12 (16)] in women who were apparently unaffected by GDM. The estimated beta coefficient from the linear regression model was 0.17 (95% CI 0.06-0.28). We performed the same analysis in women without a clinical diagnosis of GDM in the BiB study, and the estimates were very similar to the POP study: 0.13 (0.06-0.19) in BiB 1, 0.20 (0.15-0.26) in BiB 2, and 0.17 (0.13-0.21) when BiB 1 and BiB 2 were pooled.

## Discussion

The main conclusion of this analysis is that 4 metabolites in the mother’s blood were independently predictive of the risk of GDM. The evidence supporting these associations was strong. The metabolites were identified through analyses of blood samples obtained at 12 and 20 wkGA. They were validated internally using a further blood sample from the POP study obtained at 28 wkGA. Subsequently, all 4 metabolites were externally validated in 2 subgroups from a separate study (ie, BiB). Finally, we demonstrated marked enhancement of clinical prediction of GDM compared with a currently employed risk factor. A recent systematic review of metabolomic studies in GDM (24) reported a number of previous studies, but these lacked the methodological strengths of this analysis [see Supplemental Panel 2 (16)]. The generalizability of the findings is underlined by the fact that the population used for external validation was demographically highly dissimilar from the POP study. External validation was all the more remarkable due to technical variation. First, the samples studied were analyzed in 3 distinct batches, and second, the POP study samples were all nonfasting serum whereas the BiB studies were fasting plasma.

The specific metabolites identified by the present study have variable background information in relation to GDM. Mannose has not, to our knowledge, previously been associated with GDM in other studies; however, it has previously been associated with diabetes mellitus outside of pregnancy (25). 4-Hydroxyglutamate has previously been identified as a risk factor for preeclampsia in the POP study (21) but has not been associated with GDM in other studies. In contrast, there is an extensive literature on 1,5-anhydroglucitol and diabetes, both gestational and outside of pregnancy (24, 26). Lactosyl-N-palmitoyl-sphingosine (d18:1/16:0) is a lactosylceramide; its levels have previously been associated with genetic variants of the glucokinase regulatory protein, and higher levels have been associated with increased insulin sensitivity (27). However, we are not aware of any other studies demonstrating associations with any form of diabetes mellitus. In a recent study, we screened a range of obstetric complications for the presence of a metabolomic signature, using the BiB study for discovery and the POP study for validation. The 81 metabolites that were associated with GDM included the 4 metabolites selected for prediction in the present study (28). However, the aim of the present study was to identify a small panel of the most informative metabolites that may have potential utility in clinical testing. A further interesting element of the study was that the same metabolites were also predictive of birth weight among women who were not diagnosed with GDM and it may be that they also have utility in identifying women at increased risk of the complications associated with GDM but who screen negative using the current diagnostic approach.

The clinical importance of the current work is that we have identified a better method to screen women who are at high risk of GDM than BMI, which is widely employed. The findings reported indicate a potential approach to increase the sensitivity and specificity of screening for GDM. In the United Kingdom, women routinely have blood obtained around 28 wkGA to check full blood count and red cell antibodies. Hence, this test could be incorporated into practice without requiring additional phlebotomy. However, a weakness of the current study is that the assays employed were discovery metabolomics, which reports metabolites in terms of relative quantification. Actual clinical deployment of this test will require further work to generate clinical validated (eg, CE marked) assays. The development of clinical assays and their validation in other populations will be an important area of future work. However, it is also possible that optimally performed assays might improve predictive performance if there is an element of misclassification in the present study due to using relative quantitation. A further relative weakness in the present analysis is that the POP study and the BiB study employed different approaches to screening for GDM. During the period of the POP study, the hospital screened for GDM using a nonfasting 50-g GCT, and the BiB study offered all women a 75-g fasting OGTT. However, the consistency of findings across the cohorts, despite the differences, suggests that the associations are robust.

The timing of diagnostic testing for GDM in both studies followed the UK and international guidelines, and therefore the primary focus was screening at 24 to 28 wkGA. Our previous study indicated that there is a case to consider earlier screening for GDM (14). It is also possible to diagnose GDM after the routine screening at 24 to 28 wkGA, for example, when diagnostic testing is indicated by detection of a pregnancy complication such as macrosomia or polyhydramnios. In the present study, some women whom we treated as not having GDM might have passed the diagnostic threshold had they had a diagnostic screening test later in pregnancy. Any misclassification would be random with respect to the metabolite levels. As such, the expectation would be that the misclassification would bias results toward the null (ie, the reported associations might be underestimated).

There are multiple ways to analyze high-dimensional data, including, for example, regularization methods such as least absolute shrinkage and selection operator (LASSO) and elastic net regressions and tree-based classifiers such as random forests. The optimal method depends on the research question, and our aim was to generate a predictive test. We used a longitudinal mixed model approach followed by multiple testing correction to select potential candidate metabolites. Highly correlated metabolites were dropped during stepwise regression as a given metabolite would have added little predictive utility to a model that already contained a highly correlated metabolite. Internal validation used metabolite measurements at a different gestational age from the same cohort to show the extent to which the independently associated metabolites improved the prediction of GDM. Successful external validation in a highly dissimilar cohort demonstrates that our approach was robust and pragmatic.

We conclude that this study represents a further example where application of metabolomic methods can identify novel associations with human disease that can be demonstrated consistently across diverse populations. As well as this approach having broader application, it may be that some of the novel predictive metabolites identified in the current study are also independently predictive of type 2 diabetes mellitus (T2DM) outside of pregnancy, given the parallels between GDM and T2DM and the higher risk of developing T2DM in women whose pregnancies were complicated with GDM.

## Data Availability

For the POP study, restrictions apply to the availability of data generated or analyzed during this study to preserve patient confidentiality or because they were used under license. The corresponding authors will on request detail the restrictions and any conditions under which access to some data may be provided. Data requests on the POP study data can be made to the corresponding authors. Data from the BiB study are available, with very limited restriction, on request to the BiB Executive via forms that are available on the website, where information about the study and summaries of variables can also be found (https://borninbradford.nhs.uk/research/how-to-access-data/). Statistical analysis plan for external validation is available in Supplemental Panel 1 (16).
